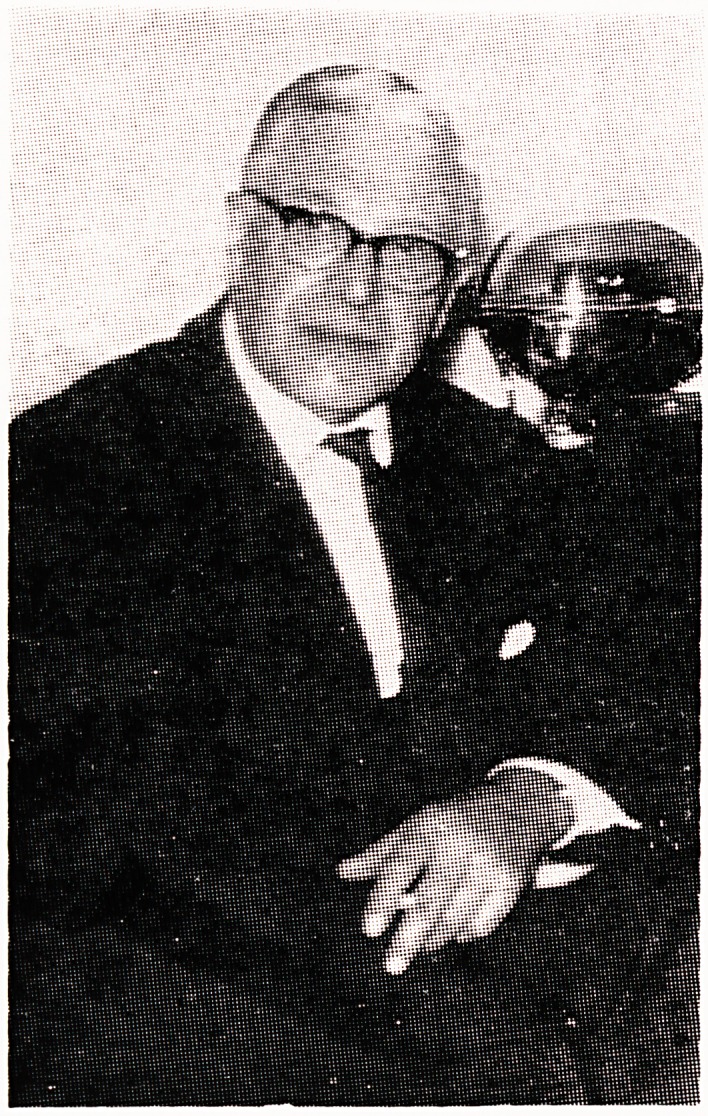# A. P. Gorham

**Published:** 1971-07

**Authors:** 


### Obituary

### A. P. GORHAM. M.B., CH.B., F.F.A.R.C.S., C.St.J.

Dr. A. P. Gorham, formerly consultant anaesthetist to the United Hospitals, Bristol, died on 6 April, 1970, at the age of 66.

Arthur Percy Gorham was born in Bristol on 10 May, 1903, and was educated at Bristol Grammar School and Bristol University, graduating M.B., Ch.B. in 1926 and taking the Conjoint diploma in the same year. After house appointments, he went into general practice and simultaneously began what was to be a very long association with the Bristol Royal Hospital for Sick Children, and with the General Hospital. During the second world war he became an anaesthetist in the Emergency Medical Service. He obtained the D.A. in 1935 and became F.F.A.R.C.S. in 1952.

Besides being consultant anaesthetist to the United Bristol Hospitals, he was clinical teacher in anaesthesia and lecturer in first-aid to Bristol University. He was awarded the rank of Commander of the Order of St. John, and edited Warwick Tunstall's "First Aid to the Injured and Sick". He also contributed papers to the anaesthetic journals. For many years, from his early days in general practice, he was a very active police surgeon. His death is a great loss to the speciality of anaesthesia in Bristol and the South-West, where he had played a prominent part in its development.

Younger anaesthetists owe an enormous debt to their predecessors of Dr. Gorham's generation who, in the face of every difficulty established the speciality in its modern flourishing state. Now that, as Professor Mushin has recently observed, "science is displacing art in British anaesthesia", the passing of these pioneers makes that displacement more complete, and perhaps we do not yet fully appreciate the value of what is being lost.

This is not to suggest that Dr. Gorham and his contemporaries were reactionary or obscurantist: on the contrary they welcomed and prudently contributed to, modern developments. But they had learnt their distinctive skills at a time when theoretical knowledge counted for relatively little, and practical artistry for so much more. Especially in anaesthetising children, Dr. Gorham had an immense experience in the simple techniques, and his safety was proverbial; he had for instance, using open ether, assisted the late Mr. W. A. Jackman in over 200 Rammstedt operations without a fatality. During the last twenty years he regularly demonstrated, to the surprise of many a young trainee and to his own secret delight, what truly excellent results are obtainable by a real expert with simple methods.

Dr. Gorham's dignified Pickwickian demeanour impressively combined solid reliability and good-mannered kindness, and it was a joy to witness him using these attributes, simply by the manner of his approach to the bed or trolley, to soothe with astonishing swiftness the apprehensions of the most nervous child. This was no mere trick, but sprang from the informed compassion of a wise doctor, a former general practitioner, who took a broad view of his function, seeing it in relation to the overall situation of each individual patient.

By precept and example Dr. Gorham inculcated in young doctors and nurses a proper pride in their professions, treating even the most junior with the respect that is their due. Many former house-officers and registrars gratefully recall, in addition to his teaching, his genuine personal interest and help in their careers. To the surgeons with whom he worked he was deferential without being obsequious; for, while recognizing the serious importance of his speciality, he had the humility to recognize also its undeniably ancillary nature.

His talents were seen to outstanding advantage in his work for the Society of Anaesthetists of the South Western Region, and was its Honorary Secretary at a particularly important time, when the initial enthusiasm might have waned. By contrast, things went from strength to strength, and Dr. Gorham was in no small measure responsible for this, for, while preserving the friendly character and tone of the Society, he established and consolidated a business-like efficiency in organization and procedure, if some thought his ideas a trifle pedantic and fussy, the years have proved their value, and the continuing success of the Society was something in which he took a modest but entirely justified pride. He was president of the Society in 1953 and it was characteristic of him that, as recently as 1964, when he could well have pleaded that he had more than done his share, he readily volunteered, during the illness of the Honorary Secretary, to assume that arduous task once more. To every newcomer he extended the same friendly welcome and helpfulness, and at the time of his retirement in July, 1968, the Society made him an Honorary Member in token of the high admiration and affection in which he was held.

Dr. Gorham was wonderfully supported throughout his full and happy career by his devoted wife, Joan, who survives him with their three children. One of their sons is a dentist, the other a doctor.

A large congregation attended the dignified and impressive Memorial Service, conducted by Dr. Gorham's lifelong friend, the Dean, at Bristol Cathedral on 9th April, 1970.

## Figures and Tables

**Figure f1:**